# Robust quantification of orientation selectivity and direction selectivity

**DOI:** 10.3389/fncir.2014.00092

**Published:** 2014-08-06

**Authors:** Mark Mazurek, Marisa Kager, Stephen D. Van Hooser

**Affiliations:** ^1^Department of Biology, Metropolitan State University of DenverDenver, CO, USA; ^2^InSPIRE Program, Concord AcademyConcord, MA, USA; ^3^Department of Biology, Brandeis UniversityWaltham, MA, USA

**Keywords:** Monte Carlo, sampling, neural data analysis

## Abstract

Neurons in the visual cortex of all examined mammals exhibit orientation or direction tuning. New imaging techniques are allowing the circuit mechanisms underlying orientation and direction selectivity to be studied with clarity that was not possible a decade ago. However, these new techniques bring new challenges: robust quantitative measurements are needed to evaluate the findings from these studies, which can involve thousands of cells of varying response strength. Here we show that traditional measures of selectivity such as the orientation index (*OI*) and direction index (*DI*) are poorly suited for quantitative evaluation of orientation and direction tuning. We explore several alternative methods for quantifying tuning and for addressing a variety of questions that arise in studies on orientation- and direction-tuned cells and cell populations. We provide recommendations for which methods are best suited to which applications and we offer tips for avoiding potential pitfalls in applying these methods. Our goal is to supply a solid quantitative foundation for studies involving orientation and direction tuning.

## Introduction

In the visual cortex of all examined mammalian species (Hubel and Wiesel, 1959, 1962), many neurons respond strongly to bars or edges at a particular preferred orientation. In some mammals such as carnivores, primates, and tree shrews, these orientation-selective cells are organized into functional columns (Hubel and Wiesel, 1962, 1968; Humphrey and Norton, 1980), and in other animals such as rodents there are no maps of orientation selectivity yet individual cells exhibit strong orientation selectivity (Girman et al., 1999; Ohki et al., 2005; Van Hooser et al., 2005; Mrsic-Flogel et al., 2007). Further, a substantial subset of orientation-selective cells also exhibit direction selectivity (Hubel and Wiesel, 1962; Weliky et al., 1996). That is, they respond more strongly to a properly oriented bar moving in a preferred direction as compared to any other direction. The functional organization and development of orientation- and direction-selective cells are the focus of intense current research.

A number of measures have been devised to assess the strength and significance of orientation and direction selectivity for a given cell (Henry et al., 1974; De Valois et al., 1982; Swindale, 1998; Ringach et al., 2002; Grabska-Barwinska et al., 2012). Traditionally, these techniques were applied to spike responses obtained from cells recorded extracellularly with microelectrodes. These cells were often identified as candidates for recording precisely because they exhibited some substantial selectivity to an oriented test stimulus that was employed while the investigator moved the electrode, hunting for cells.

New challenges—the advent of unbiased optical recording techniques such as 2-photon calcium imaging that sample all cells regardless of selectivity (Stosiek et al., 2003; Kerr et al., 2005; Garaschuk et al., 2006) and the need to characterize cells in developing animals with poor, emerging selectivity—have introduced new difficulties for assessing orientation and direction selectivity. Some of the traditional measures of selectivity can give noisy or spuriously high values when applied to cells that don't exhibit at least moderate selectivity. Further, exciting new molecular and circuit techniques are permitting the testing of very precise circuit hypotheses about the mechanisms underlying orientation and direction selectivity. Knowledge about statistical power—the number of cells or repetitions of a stimulus that are needed in order to observe a change in selectivity of a particular size—is critical for these studies.

Here we characterize the robustness of several measures of orientation and direction selectivity on simulated responses. We provide a recommendation for analysis methods for the principle questions that investigators usually ask: (1) How much orientation or direction selectivity does a cell exhibit? (2) Does a cell exhibit significant orientation or direction selectivity? (3) Has a manipulation introduced a significant change in the amount of orientation or direction selectivity at the population level? Further, we provide tables for statistical power, to estimate the amount of data that would be required to accurately answer these questions. These methods could in principle be extended to other sensory response properties or other modalities; however, their performance depends on the form of the underlying response function, so they may perform less reliably in domains aside from orientation and direction selectivity.

## Materials and methods

### Coordinate systems

There is no standard coordinate system for indicating orientation or direction space. In this paper, we use “compass” coordinates, in which a horizontal bar moving upward is considered to be moving at 0°, and angles increase in a clockwise manner. Another common coordinate system is the Cartesian system, where 0° indicates a vertical bar moving to the right, and angles increase in a counterclockwise direction. One can transform between these two systems using the following equations:

$$\begin{array}{l} \theta_{cartesian}=90^{{}^{\circ}}-\theta_{compass}\\ \theta_{compass}=90^{{}^{\circ}}-\theta_{cartesian} \end{array}$$

Because there is no standard coordinate system for orientation and direction, and because some readers may be unfamiliar with orientation and direction, it is helpful to use pictures to indicate stimulus orientation and direction in slides and in published figures, as we do here.

### Angular addition

In several equations, we express angles in terms of the sum of angles. For example, for a direction tuning curve we define the positive orthogonal orientation as follows: θ_*orth*+_ = θ_*pref*_ + 90°. Note that these angles are summed modulo 360° in direction space and modulo 180° in orientation space. For example, in direction space, 359° + 2° = 361° modulo 360° = 1°.

### Relationship of *OI* and *DI* to other commonly used measures of orientation and direction selectivity

In this paper, we use *OI* and *DI* as the normalized measures of “peak to trough” orientation selectivity and direction selectivity (see “Results”): *OI* = (*R_pref_ori_* − *R_orth_*)/*R_pref_ori_* and *DI* = (*R_pref_* − *R_null_*)/*R_pref._* Many papers use a slightly modified version of these measures that we will call the OSI and DSI: *OSI* = (*R_pref_ori_*− *R_orth_*)/(*R_pref_ori_*+ *R_orth_*) and *DSI* = (*R_pref_*−*R_null_*)/(*R_pref_* + *R_null_*). Still other papers use the “orthogonal to peak” ratio to quantify orientation selectivity: *O*/*P* = *R_orth_*/*R_pref_ori_* = 1 − *OI*. In making quantitative comparisons across papers, the reader should note carefully which is being used.

The response at the preferred orientation *R_pref_ori_* and the response at the preferred direction *R_pref_* can be determined in different ways. In some measures, these are taken to be the best response to one of the stimulus orientations or directions that was explicitly measured; that is, if we measure responses at stimulus directions θ_1_, θ_2_,…,θ_*n*_, then we choose the response at the best θ_*i*_. In other measures, we perform a fit to the tuning curve, and choose the maximum value of the fit as *R_pref_ori_* or *R_pref_*.

### Monte carlo simulations

In Monte Carlo simulations of orientation and direction tuning curves, the “true” preferred angle and tuning widths were varied randomly so that a variety of tuning curve shapes were analyzed. Ranges were selected to correspond to typical values observed in V1 neurons. Each underlying “true” curve was a double Gaussian. The underlying angle preferences were chosen according to a uniform distribution between 0° and 360°. Tuning widths were chosen randomly according to a Gamma distribution with shape 3 and scale 6: σ = (Gamma(3,6)+10°)/1.18.

In many of the figures, we examined curves with 21 values of underlying *OI* or *DI*. These were produced in the double Gaussian equation (see **Results**) by setting the baseline rate *C* = 10 − (*i* − 1)/2, *R*_*p*_ = (*i* − 1)/2, *R_n_* = (*i* − 1)/4, for *i* from 1 to 21. In other figures we examined 21 values of underlying *DI*: *C* = 0, *R*_*p*_ = 10, *R_n_* = 10 − (*i* − 1)/2. Note that all of these curves with varying direction selectivity exhibit high orientation selectivity (that is, orientation selectivity and direction selectivity levels were not co-varied).

To calculate statistical power for simulated 2-condition experiments, we simulated underlying orientation or direction curves with exactly the *OI* or *DI* specified, and randomly varied the preferred angle and tuning width as described above. We simulated 100 populations of increasing sizes, and calculated the minimum size when *X*% of these simulations produced significant differences by applying a *t*-test with confidence *X*%, for *X* = 95, 99, and 99.9.

### Noise models

In each set of simulations, the simulated noise parameter is described. We used two types of noise. The most common type of noise, intended to capture the statistics of spikes recorded with an extracellular electrode, was a constant Gaussian noise value that was added to responses of all orientations on all trials. This constant value is often expressed as a percentage of the maximum response, which was usually 10 Hz. So, 20% noise means 2 Hz noise was added to individual trial measurements.

A more recent technique for recording neural responses is 2-photon imaging with Oregon Green BAPTA-1 AM (OGB-1AM) (Stosiek et al., 2003; Ohki et al., 2005; Garaschuk et al., 2006). This method involves bulk loading of neural tissue with a calcium indicator bound to an AM-ester, leading to the uptake of the calcium indicator by all neurons within the loading region. This technique produces noise characteristics that differ from extracellular spike recordings. Specifically, because 2-photon calcium imaging records intracellular calcium concentration rather than membrane voltage, it tends to show a lower signal-to-noise ratio and a constant background signal. By examining the responses in previous experiments (Li et al., 2008), we modeled this noise as Gaussian noise with magnitude equal to a constant factor plus a component that depended on the response at each direction, such that noise = 20% + 10% ^*^ response magnitude.

## Results

Orientation selectivity has been observed in the visual cortex of every mammal that has been examined, including carnivores (Hubel and Wiesel, 1962), primates (Hubel and Wiesel, 1968), rodents, including murid (Girman et al., 1999; Niell and Stryker, 2008), and sciurid rodents (Heimel et al., 2005; Van Hooser et al., 2005), and marsupials (Rocha-Miranda et al., 1976; Ibbotson and Mark, 2003).

**Figure 1:**
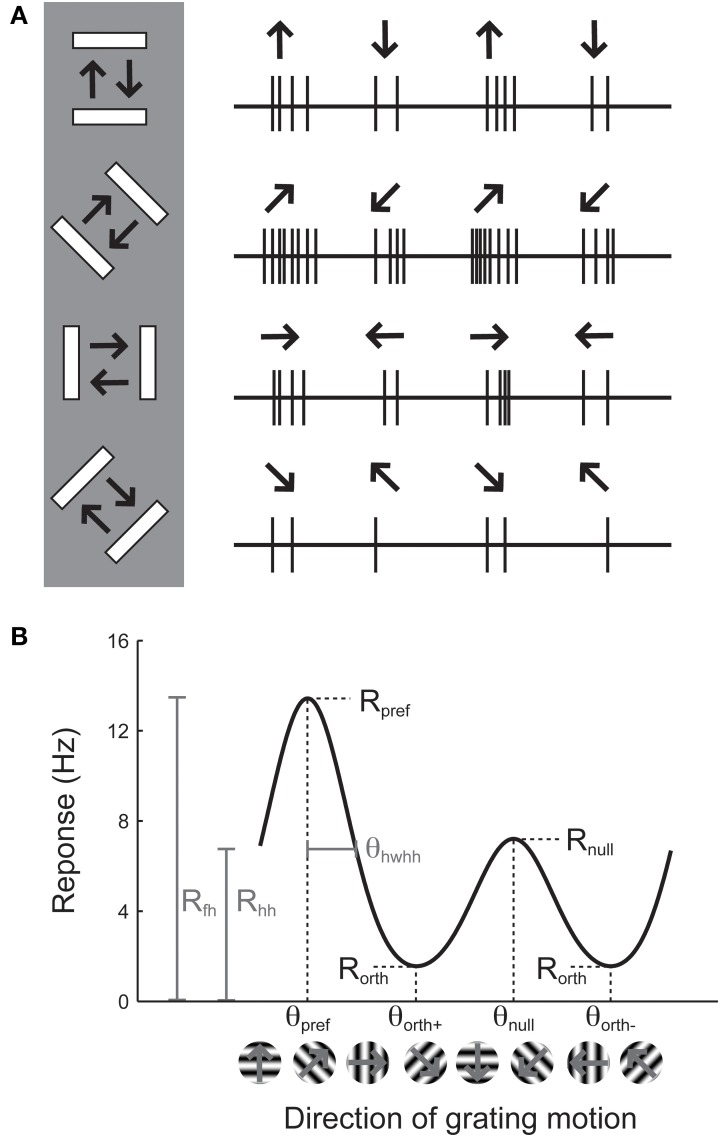
**Illustration of the assessment of orientation and direction selectivity. (A)** Left: Depiction of a bar stimulus moving at different orientations across the receptive field of an example cell. The cell's responses to each orientation are indicated at the right. The preferred orientation is 45°. During each presentation of the bar stimulus, the stimulus moves back and forth in two opposite directions. This cell responds more strongly to movement of the bar toward 45° than it does to the opposite direction (225°). **(B)** A graph of responses to the same cell to sinusoidal gratings drifting in several directions. The cell gives the largest response (*R_pref_*) to 45° (θ_*pref*_), and a weaker response (*R_null_*) to the opposite direction 225° (θ_*null*_). The cell responds less strongly to stimulation at either of the two orthogonal orientations (θ_*orth*+_ and θ_*orth*+_). The cell's response decreases as the direction of the stimulus deviates from θ_*pref*_; the difference between θ_*pref*_ and the angle that causes the response to drop to half (*R_hh_*) its maximum value is called the half width at half height (θ_*hwhh*_).

Orientation selectivity is traditionally assessed by sweeping a bar or by drifting sinusoidal gratings across the cell's receptive field in different directions (Hubel and Wiesel, 1962; Movshon et al., 1978, pp.101–120), although it can also be assessed by flashing static bars at different orientations (Palmer and Davis, 1981). The example cell in Figure 1A exhibits a substantial response to bars that are rotated at a 45° angle from horizontal. The stimulus in Figure 1A moves in two opposite directions. The response to upward and rightward motion (45°) is stronger than the response to downward and leftward motion (225°), indicating that the cell is sensitive not only to the orientation of the stimulus but also its direction of motion. Note that every cell that is direction-selective by this criterion is also orientation-selective, but an orientation-selective cell is not necessarily direction-selective, because a cell could exhibit equal responses to the two opposite directions.

The responses of this example cell are shown on a graph in Figure 1B. Each response is presented as a firing rate: the number of spikes evoked by each stimulus has been divided by the duration of the stimulus in seconds. We imagine that the experimenter has collected some responses to “blank” stimuli, where the screen remains blank for the same time duration as for each bar stimulus, and has subtracted these “blank” or “background” responses from each measurement so that we are examining the contribution of the oriented stimulus to the cell's firing rate and not ongoing background activity. This collection of responses to a set of different orientations or directions is called an “orientation tuning curve” or a “direction tuning curve,” respectively. The major descriptive features of orientation and direction tuning curves are illustrated. The stimulus angle that evokes the maximum response *R_pref_* is called the preferred direction (θ_*pref*_), while the opposite direction is called the null direction (θ_*pref*_ +180°).

### Quantifying the degree of orientation and direction selectivity

From the graphical tuning curve in Figure 1B, it is easy to imagine two major notions of orientation selectivity. One is a comparison of the cell's response to the preferred orientations (*R_pref_* + *R_null_*) compared to the responses (*R*_*orth*+_ and *R*_*orth*−_) at the orientations that are orthogonal (θ_*orth*+_ = θ_*pref*_ + 90°, θ_*orth*−_ = θ_*pref*_ − 90°) to the preferred orientation. This method has been employed in numerous studies, and we refer to it here as the *orientation index* (*OI*):

$$OI=(R_{pref}+R_{null}-\left(R_{orth+}+R_{orth-}\right))/\left(R_{pref}+R_{null}\right)$$

Note that it is not necessary to stimulate with directional stimuli in order to obtain a measure of orientation selectivity. Indeed, in many studies, the bars are drifted back and forth and the responses to each pair of opposite directions are averaged together. In this “orientation space,” the angle of stimulation ranges from 0° to 180°. We can calculate the orientation selectivity index in this case by using the preferred response (*R_pref_ori_*) and the response *R_orth_* at the orthogonal orientation (θ_*orth*_ = θ_*pref*_ + 90°)

$$OI=(R_{pref\_ori}-R_{orth})/R_{pref\_ori}.$$

The *OI* can nominally vary from 0 (no selectivity) to 1 (perfect selectivity), although it can exceed 1 if the response to the orthogonal orientation drops below the background firing rate, that is, when *R_orth_* is negative.

In direction space, a *direction index* can be defined similarly:

$$DI=\left(R_{pref}-R_{null}\right)/R_{pref.}$$

Another major notion of orientation or direction selectivity is the sensitivity of the response to the preferred angle. One can imagine measuring the amount one needs to change the orientation (or direction) angle from the preferred for the response to drop by some amount, such as by half (*R_hh_*, the response at half-height). The angle difference θ_*hwhh*_ indicates how far in orientation space one must adjust the angle from the optimal to obtain half of the response height. This type of selectivity has been referred to as a cell's *orientation tuning width*. Measuring the orientation tuning width requires either substantial sampling of responses to different angles, or performing a tuning curve fit, which we will turn to later.

**Figure 2:**
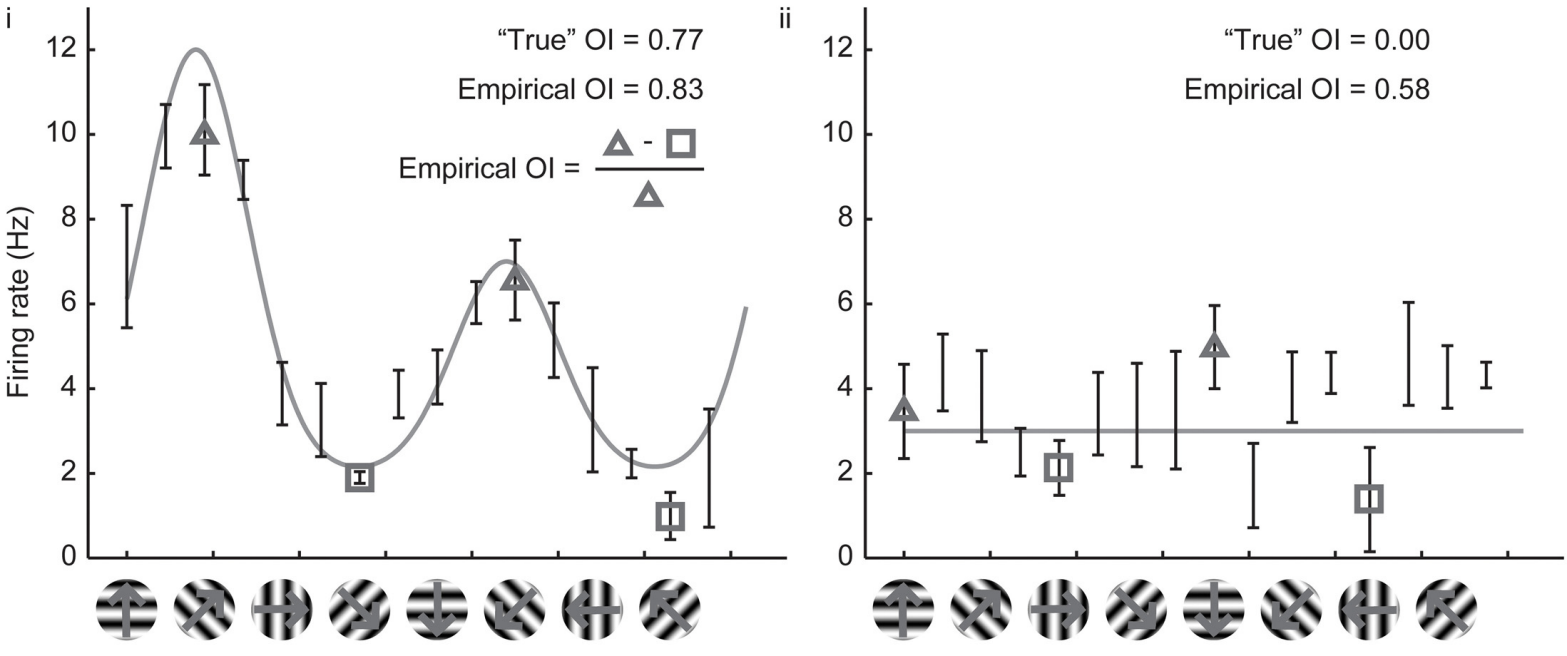
**Calculation of the empirical orientation selectivity index (*OI*). (i)** Simulated responses (10 trials, 5 Hz per trial noise) to a model cell with an underlying direction tuning curve indicated in gray. Error bars indicate standard error around the mean of the simulated responses. To calculate *OI*, responses from the preferred orientation angle are averaged together (triangles) and the responses to the orthogonal angles (squares) are subtracted. This quantity is normalized by the response to the preferred orientation angle (triangles). There is good qualitative agreement between the empirical *OI* and the “true” *OI*. **(ii)** Same, for a model cell that is not orientation selective. The empirical *OI* is still very large due to the noise in the simulated responses, and is not qualitatively similar to the “true” underlying *OI*.

Owing to the mathematical simplicity of the *OI*, most studies over the years have employed the *OI* to assess orientation selectivity. By ear, it is easy to assess whether or not a cell exhibits perfect orientation selectivity with the *OI* (that is, when there are no responses to the orthogonal angle), and the *OI* is very easy to compute numerically even when one is measuring spikes or firing rates. When applied to cells with substantial selectivity, it provides a simple and valid measure of orientation selectivity. Figure 2 shows simulated responses from a model neuron (**i**) with a theoretical or “true” *OI* of 0.77; we have simulated 10 repeated trials of each direction; 5 Hz of random noise was added to each simulated trial, and the means are plotted. The empirical *OI*, measured by taking the average of the responses at the preferred orientation subtracting the responses at the orthogonal orientation, and normalizing, we obtain a value (0.83) very close to the “true” *OI* value of 0.77.

However, the case of a model cell (**ii**) with no orientation selectivity is also shown in Figure 2. Again, we have simulated 10 trials with 5 Hz of added noise. If we blindly report the empirical measure of *OI*, we obtain a value 0.58, a value much larger than the “true” value of 0. The reason is that we always choose the angle of the empirical maximum response to be “preferred,” and, in this case, that angle was just the angle that had the largest response due to noise only (there was no orientation signal). By random chance in this example, the responses at the angles that correspond to the orthogonal orientations (represented by the squares) are both less than the responses to the preferred orientation (represented by the triangles), so the *OI* is large.

In single unit recording studies in adult animals, one often ignores cells with weak responses, but if one is conducting an imaging study of 100's of neurons, or developmental research in animals with weakly responsive cells, it is highly likely that some neurons will exhibit weak orientation selectivity. If the *OI* is applied blindly, it is likely that many of these weakly selective neurons will have empirical *OI* values that are high, only due to noise.

### Vector spaces for orientation and direction

**Figure 3:**
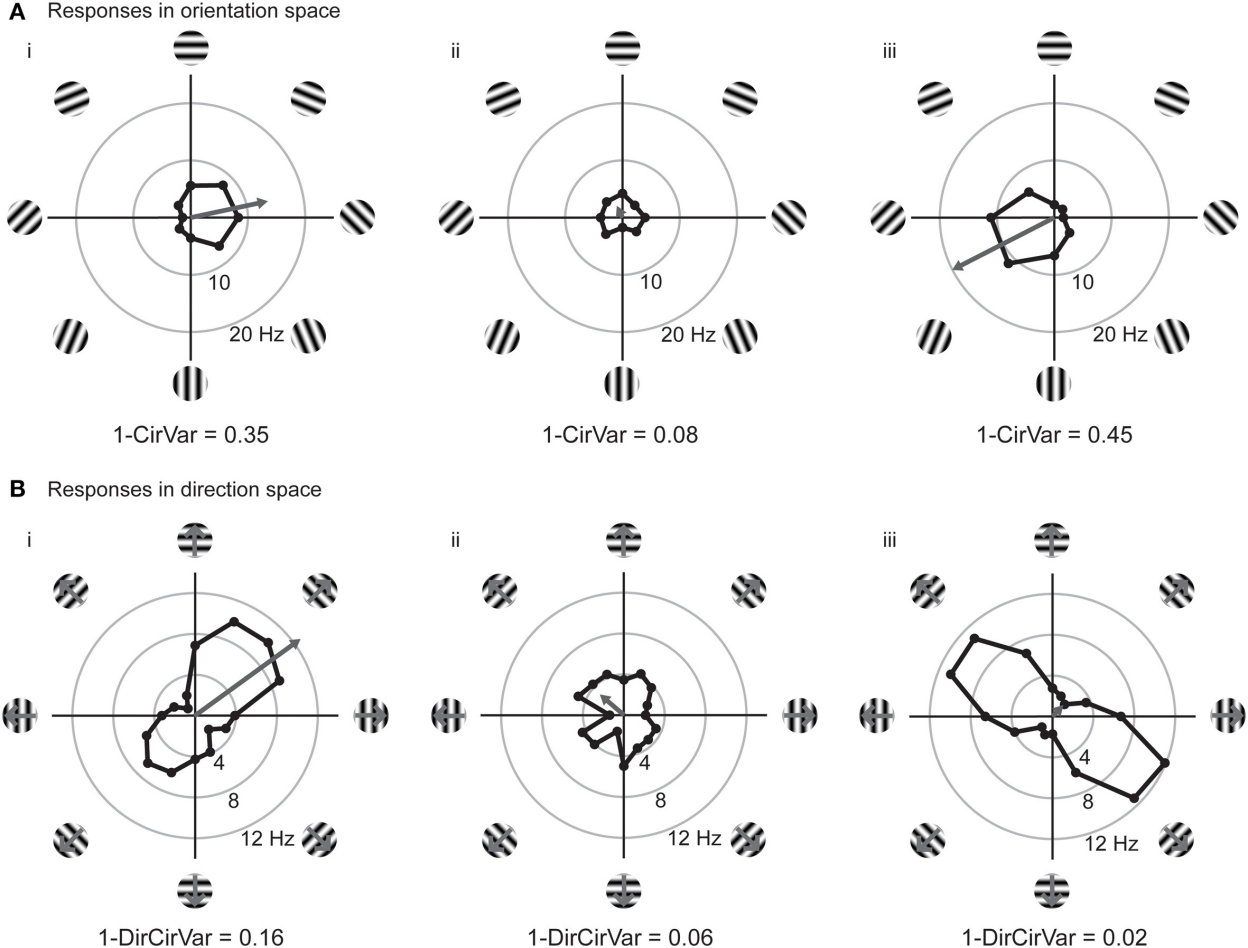
**Responses of model cells in polar coordinates on the complex plane**. **(A)** Responses of model cells in orientation space. Response (in spikes per second) at each angle is indicated by the distance from the origin. Orientation angles vary from 0 (horizontal) to 90° (vertical) and back to 0°/180° (horizontal). Gray arrow indicates the vector mean of the responses to individual orientations. The normalized length of the mean response vector is the quantity *L_ori_*, which is 1 minus the circular variance (1-*CirVar*). Model cells **i** and **ii** are the same as in Figure 2. **(B)** Responses of the same cells plotted in direction space. Direction of motion is indicated, response at each angle is indicated by the distance from the origin. Note that 0° and 180° both correspond to horizontal stimulus orientations, but moving in upward and downward directions, respectively. Gray arrow indicates the vector mean of responses to individual directions. Note that cell **iii** is highly orientation selective for oblique bars but is poorly selective for stimulus direction.

We can improve the situation by plotting the responses to individual stimuli in a vector space. In Figure 3, we have replotted the responses of model cells (**i**,**ii**) and a new model cell (**iii**) in polar plots. Figure 3A shows the responses plotted in orientation space, where values for responses to the two opposite directions have been averaged, and angles vary from 0° and 180°. Figure 3B shows the responses plotted in direction space, where angles vary from 0° to 360°. The graphs also show, in gray, the vector that is the sum of all of the mean responses in vector space. In these examples, the length of the vector sum is more related to the amount of orientation selectivity as compared to the *OI* index.

The normalized length of this vector in orientation space is computed as follows:

$$L_{ori}=\left|\frac{\sum_{k}R(\theta_{k})\exp(2i\theta_{k})}{\sum_{k}R(\theta_{k})}\right|,$$

where *R*(θ_*k*_) is the response to angle θ_*k*_. In direction space this length is the following:

$$L_{dir}=\left|\frac{\sum_{k}R(\theta_{k})\exp(i\theta_{k})}{\sum_{k}R(\theta_{k})}\right|.$$

The normalized vector length is related to a classic quantity in circular statistics called the *circular variance* (Batschelet, 1981; Ringach et al., 2002):

$$1-CirVar=L_{ori}$$

We use the abbreviation *CirVar* to differentiate the circular variance from the classic statistical quantity called “coefficient of variation,” which is often abbreviated as CV. We similarly define a quantity called 1-*DirCirVar*:

$$1-DirCirVar=L_{dir}$$

This definition differs (by a factor of 2) from the classic definition of circular variance in direction space (Batschelet, 1981), but we leave off the factor of 2 here so that a cell with maximal selectivity has a 1-*DirCirVar* of 1 (rather than 2).

The vector lengths in orientation space (1-*CirVar*) and in direction space (1-*DirCirVar*) for the model cells **i**–**iii** are shown in Figure 3. There are two important things to notice in comparison to the *OI*. First, model cell **ii** has a high (spurious) empirical *OI* value (Figure 2ii) but has a small 1-*CirVar* value, indicating that 1-*CirVar* is closer to the true selectivity of the cell, which is 0. Second, a cell can only have a 1-*CirVar* value of 1 when it exhibits a response to 1 orientation and no other orientations. Cells with high selectivity that would have *OI* values near 1, such as model cells **i** and **iii**, generally have lower 1-*CirVar* values, since cells typically respond to more than a single orientation; that is, the response tuning generally has some width. Thus, circular variance depends on both selectivity and tuning width.

### Comparing *OI* and circular variance (and *DI* and direction circular variance)

When one records a neuron experimentally, one can only obtain a limited number of samples of the neuron's responses. One would like to use these sampled responses to make the best guess about the neuron's “true” properties, which cannot be examined directly but can only be inferred from experimental observations. Here we used Monte Carlo simulations to consider which index, *OI* or circular variance, allows one to make the best guess about the true orientation selectivity.

**Figure 4:**
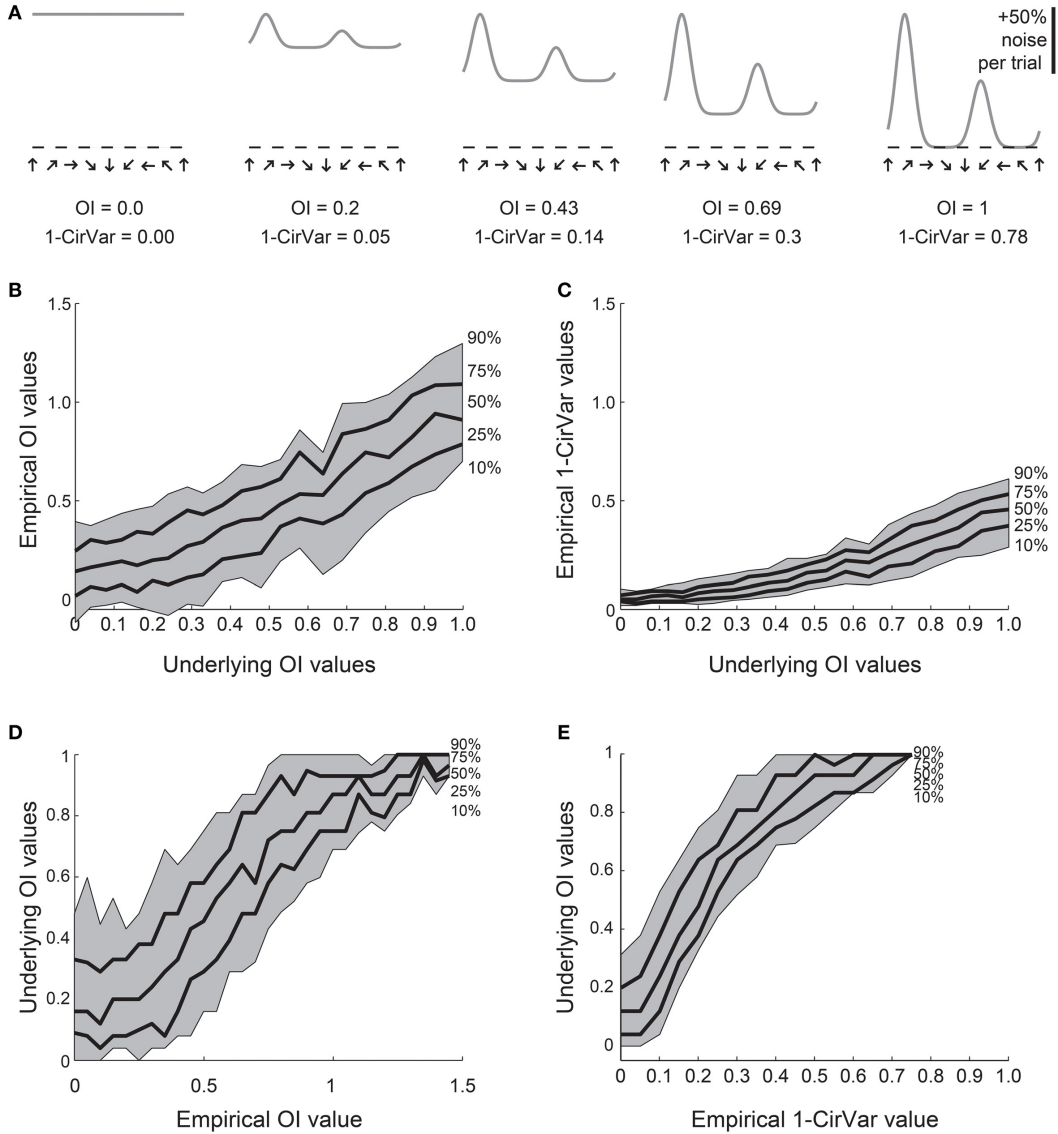
**Comparison of *OI* and circular variance measures for simulated data**. We created 100 simulations of tuning curves for each of 21 underlying “true” tuning curves, ranging from *OI* = 0 to *OI* = 1, some shown in **(A)**. Each trial had 50% noise added. **(B)** Percentiles of the empirically determined *OI* for the 100 simulations at each underlying “true” *OI* value. Note that for cells with 0 true selectivity, the empirical *OI* values range from slightly negative to almost 0.5. **(C)** Percentiles of the empirically determined 1-*CirVar* index for each of the underlying “true” *OI* values. Note that when “true” *OI* is low, the 1-*CirVar* is always low. The index 1-*CirVar* increases as “true” *OI* increases but the range of values remains narrower than the corresponding range of empirical *OI* values in **(B)**. **(D)** The inverse of **(B)**; given we observed an empirical *OI* value of x, what is the range of possible “true” *OI* values that produced x in our simulations? An empirical *OI* of 0 could have arisen from cells with “true” *OI* values ranging from 0 to 0.5, and an empirical *OI* of 0.5 could have arisen from cells with a “true” *OI* ranging from about 0.1 to about 0.8. **(E)** The inverse of **(C)**. A 1-*CirVar* of 0 could have arisen from a “true” *OI* ranging from 0 to about 0.3, and 1-*CirVar* of 0.25 could have arisen from a “true” *OI* ranging from about 0.4 to 0.8. The range of possible underlying “true” *OI* values is much narrower when 1-*CirVar* is used as a readout as compared to *OI*.

We created 21 model orientation tuning curves that ranged in “true” selectivity from 0 to 1 (Figure 4A). From each model, we simulated 100 tuning curves with 10 experimental trials each by adding 50% single trial noise; the exact angle preference and tuning width was chosen randomly (see Materials and Methods). We then calculated the *OI* and 1-*CirVar* for each simulation (Figure 4).

The percentile distribution of empirical *OI* values for each “true” *OI* value is shown in Figure 4B. There is a wide range of empirical *OI* values; for example, when the “true” *OI* is 0, empirical *OI* values ranged from slightly negative to about 0.5. By contrast (Figure 4C), the distribution of circular variance values is much tighter; when “true” *OI* is 0, the 1-*CirVar* value is always nearly 0.

While the results in Figures 4B,C show the range of empirical *OI* and 1-*CirVar* values that one might expect for a given “true” *OI*, the most relevant relationship for experimentalists is the inverse relationship: given that one observes an empirical *OI* value of *X* or a 1-*CirVar* value of *Y*, what are the likely “true” *OI* values that could underlie these empirical values? As shown in Figure 4D, knowing the empirical *OI* value tells one very little about the “true” *OI* value: for example, if the empirical *OI* is 0, the “true” *OI* could be as high as 0.5. On the other hand (Figure 4E), the circular variance gives more information about the “true” *OI*: if we obtain an empirical 1-*CirVar* of 0, the “true” *OI* is likely to be less than 0.3; if we obtain an empirical 1-*CirVar* of 0.25, the “true” *OI* is likely to be between 0.4 and 0.7.

**Figure 5:**
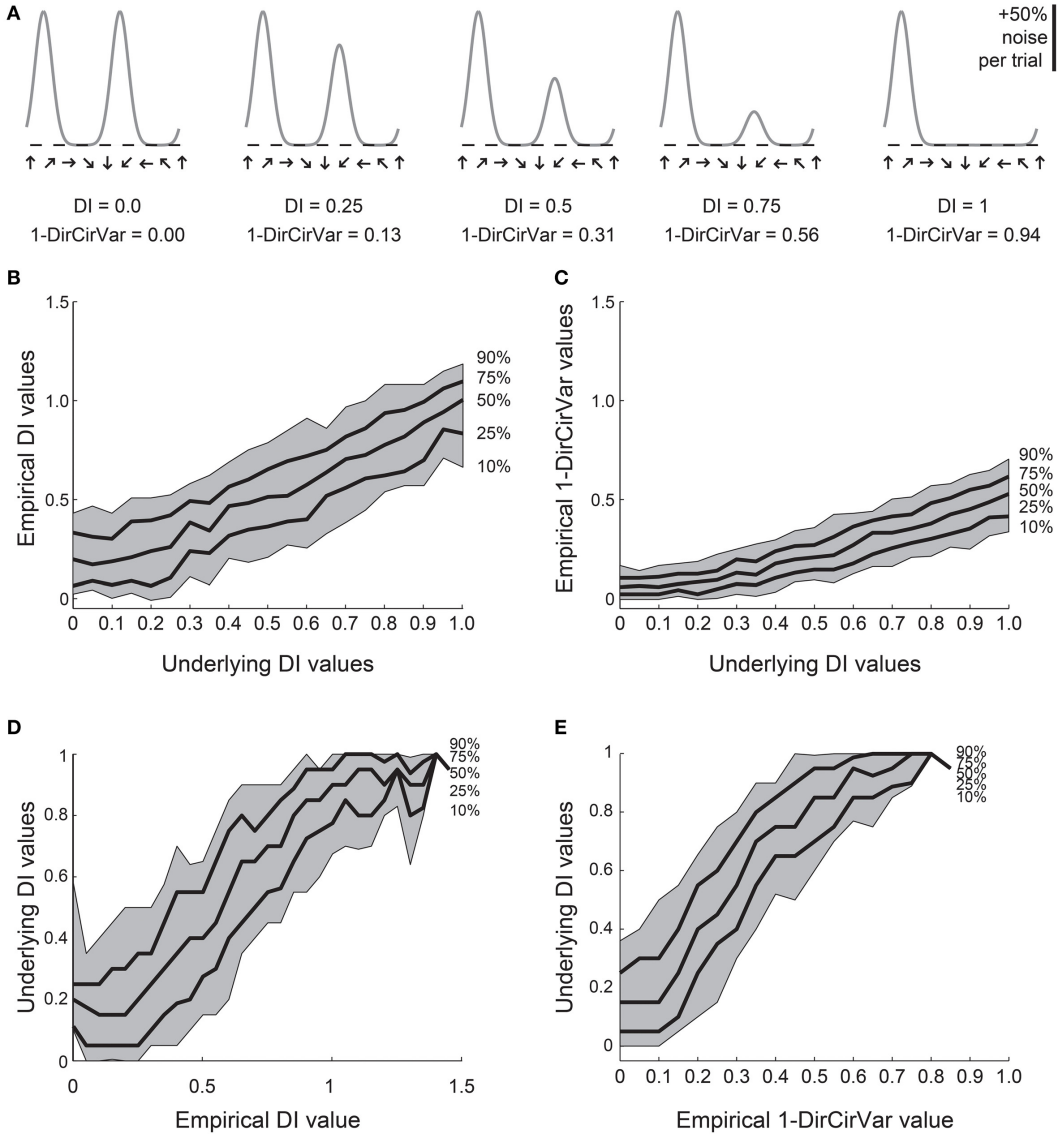
**Comparison of *DI* and direction circular variance measures for simulated data**. We created 100 simulations of tuning curves for each of 21 underlying “true” tuning curves, ranging from *DI* = 0 to *DI* = 1, some curves shown in **(A)**. Each trial had 50% noise added. **(B)** Percentiles of the empirically determined *DI* for the 100 simulations at each underlying “true” *DI* value. Note that for cells with 0 true selectivity, the empirical *DI* values range from about 0 to about 0.5. **(C)** Percentiles of the empirically determined 1-*DirCirVar* index for each of the underlying “true” *DI* values. Note that when “true” *DI* is low, the 1-*DirCirVar* is always low. The index 1-*DirCirVar* increases as “true” *OI* increases but the range of values remains narrower than the corresponding range of empirical *DI* values in **(B)**. **(D)** The inverse of **(B)**; given we observed an empirical *DI* value of x, what is the range of possible “true” *DI* values that produced x in our simulations? An empirical *DI* of 0 could have arisen from cells with “true” *DI* values ranging from about 0 to 0.5, and an empirical *DI* of 0.5 could have arisen from cells with a “true” *DI* ranging from about 0.1 to 0.7. **(E)** The inverse of **(C)**. A 1-*DirCirVar* of 0 could have arisen from a “true” *DI* ranging from 0 to about 0.4, and 1-*DirCirVar* of 0.25 could have arisen from a “true” *DI* ranging from about 0.1 to 0.7. The range of possible underlying “true” *DI* values is narrower when 1-*DirCirVar* is used as a readout as compared to *DI*.

We performed similar simulations for direction selectivity, comparing the empirical *DI* with the empirical 1-*DirCirVar* (Figure 5). The difference in uncertainty about the “true” *DI* between the *DI* and 1-*DirCirVar* is less pronounced than the difference in uncertainty between the *OI* and 1-*CirVar*, but nevertheless the empirical 1-*DirCirVar* provides more information about the “true” *DI* than the empirical *DI*.

The Monte Carlo simulation results presented in Figures 4, 5 provide strong evidence that circular variance is a more robust and reliable indicator of the amount of orientation or direction selectivity than the *OI* or *DI*. The circular variance works well when selectivity is strong or weak. We recommend the use of circular variance whenever quantification of the amount of orientation or direction selectivity is necessary.

**Figure 6:**
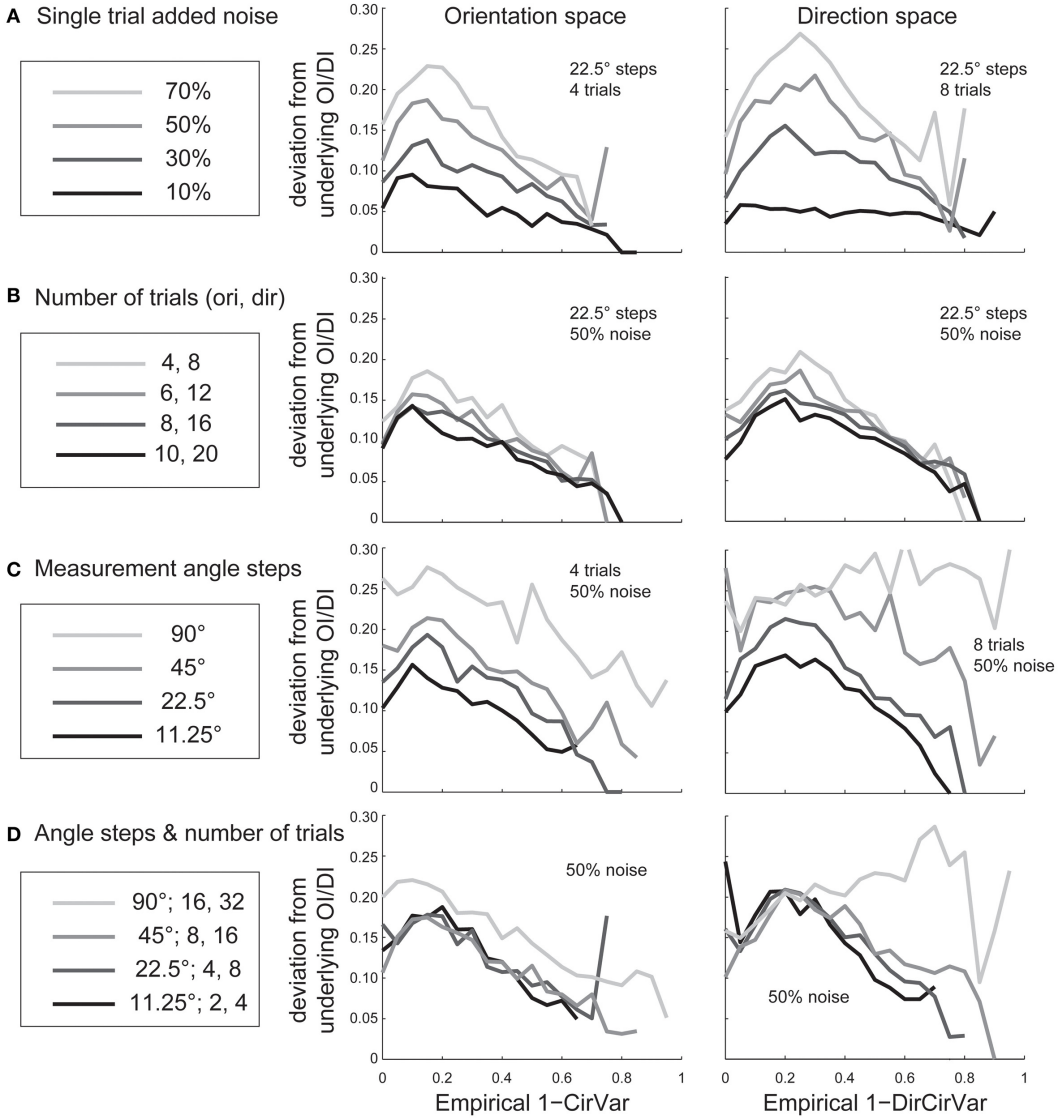
**The dependence of error in identifying the true *OI* on neural noise and stimulus sampling**. On the Y axis of all plots is the average deviation between the “true” *OI* (or *DI*) and the “best guess” of *OI* (or *DI*) based on the empirical 1-*CirVar* (or 1-*DirCirVar*). **(A)** Dependence of error on single trial noise as a percentage of the maximum response rate to the preferred direction. **(B)** Dependence of error on the number of trials. More trials offer modest improvements in average accuracy. **(C)** Dependence of error on number of angle steps. Additional angle steps offer a big improvement in estimating the amount of orientation or direction selectivity present. **(D)** Dependence of error for assorted numbers of trials and angle steps.

Experimentalists are also interested in knowing how many stimulus trials and stimulus angle steps should be presented to the animal in order to provide a quality estimate of the neuron's true orientation or direction selectivity. We performed Monte Carlo simulations where we systematically varied the single trial noise, number of stimulus trials, and the number of stimulus angles in order to understand how these factors influenced error in uncovering the “true” *OI* or *DI* (Figure 6). As expected, more trials and more angles were always better, but 45° angle steps and eight trials (or 22.5° steps with four trials) appear to be the minimum required for a quality assessment of direction selectivity. Naturally, this result depends on the reliability of the neuron being studied; neurons with lower responsiveness or higher noise will require more trials and/or stimulus angles.

### Significance of orientation and direction tuning

When one suspects a cell is selective for stimulus orientation and/or direction, it is often important to verify this selectivity statistically. We need tools that allow us to answer the question “is a cell's selectivity for orientation/direction statistically significant?” In principle, one could simply measure a selectivity coefficient on each trial and perform statistics on this distribution of coefficients. However, the flaw in this analysis is in determining the null hypothesis with selectivity coefficients, and the flaw applies whether one uses *OI*/*DI* or 1-*CirVar*/1-*DirCirVar*. Specifically, for all these coefficients, the expected value in the absence of selectivity is greater than zero because any variance across stimulus angles, whether stimulus-driven or random, always produces coefficient values greater than zero. Thus, one could not use this method to prove that a particular measured coefficient was not simply produced by noise.

**Figure 7:**
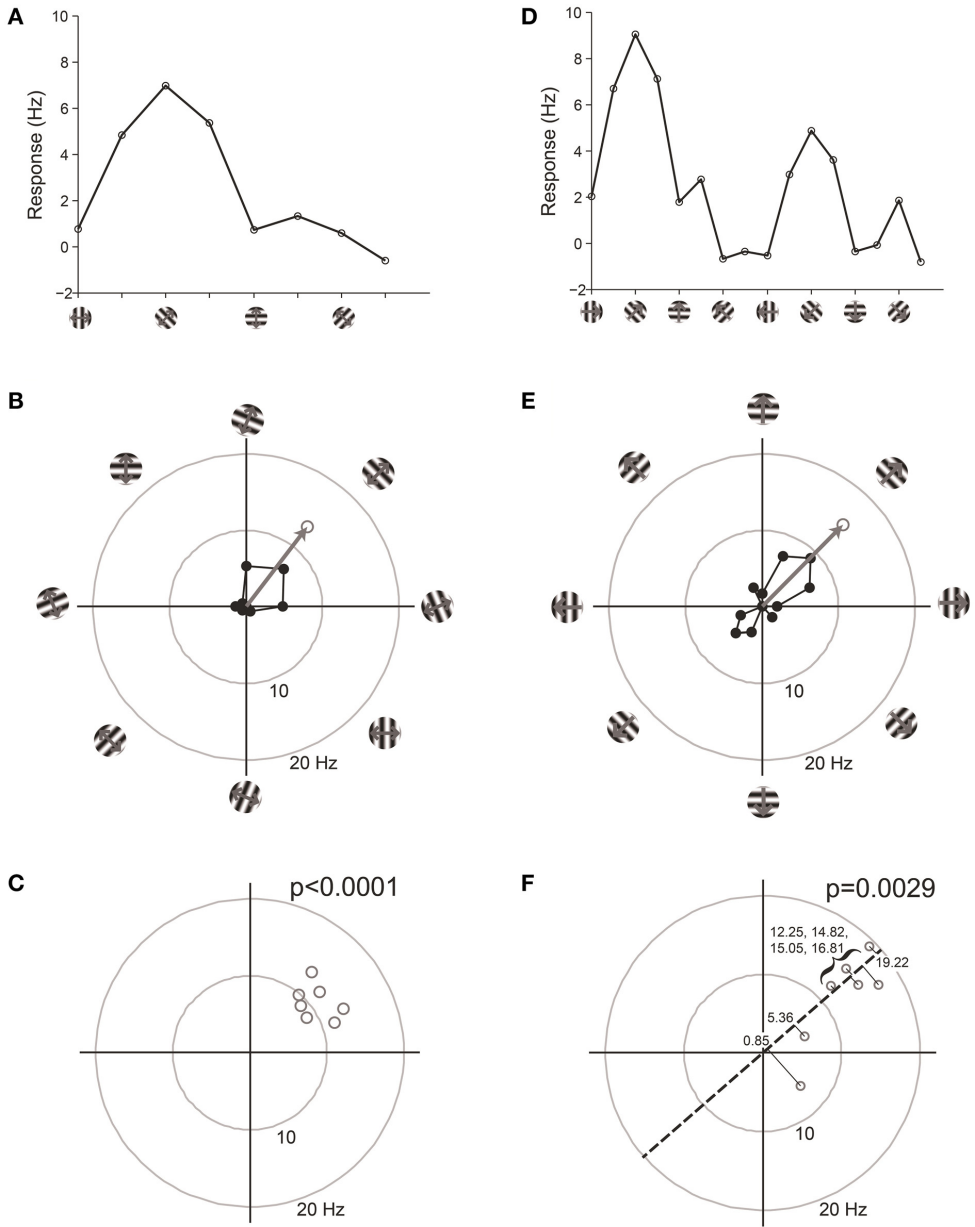
**Vector-based statistical tests with orientation (A–C) and direction (D–F) responses**. This figure uses data from a model cell with strong tuning: Underlying *OI* = 0.9, *DI* = 0.5, noise = 20%. 16 directions (22.5° steps) were tested. For the orientation analysis, opposite directions at the same orientation were averaged together. **(A)** One trial from the model cell plotted in orientation response. Note that a “trial” is defined here as one measurement at each stimulus orientation. **(B)** The response from the trial shown in **(A)**, plotted in polar coordinates. *Black:* The response obtained at individual orientations. *Gray:* The vector sum of the responses at individual orientations. This is the “orientation vector” on this trial. **(C)** Orientation vectors from seven trials from the model cell. *Gray circles* show the orientation vectors from the seven trials. The *p*-value above the graph gives the result of Hotelling's *T*^2^-test, which tests for whether the 2-dimensional mean of this distribution of orientation vectors is different from [0, 0]. **(D)** One trial from the model cell plotted in direction space. Here a “trial” is defined as one measurement at each stimulus direction. **(E)** The response from the trial shown in **(D)**, plotted in polar coordinates. *Gray:* The vector sum of the responses at individual directions. This is the “direction vector” on this trial. **(F)** Direction vectors from seven trials from the model cell. *Gray circles* show the direction vectors from the seven trials. The *dashed line* is orientation axis from this cell, obtained by measuring the angle of the average orientation vector. *Black lines* show the projection of the direction vectors onto the orientation axis (the “direction dot products”). *Numbers* give the magnitude of the direction dot product for each direction vector. The *p*-value above the graph gives the result of Student's *T*-test applied to the direction dot product values against H0: Mean = 0.

We have found that the best way to detect selectivity is to measure the magnitude of orientation or direction vectors (Figure 7). For this test, we organize data into “trials,” where a trial is one response at each stimulus orientation or direction. The orientation or direction vector is calculated on each trial as the vector sum of responses on that trial measured in orientation or direction space, respectively. The magnitude of the vector correlates with the degree of selectivity, and the expected magnitude is zero for zero selectivity. Hence we can perform statistics on the distribution of vector magnitudes against the null hypothesis H0: that the magnitude equals zero.

**Figure 8:**
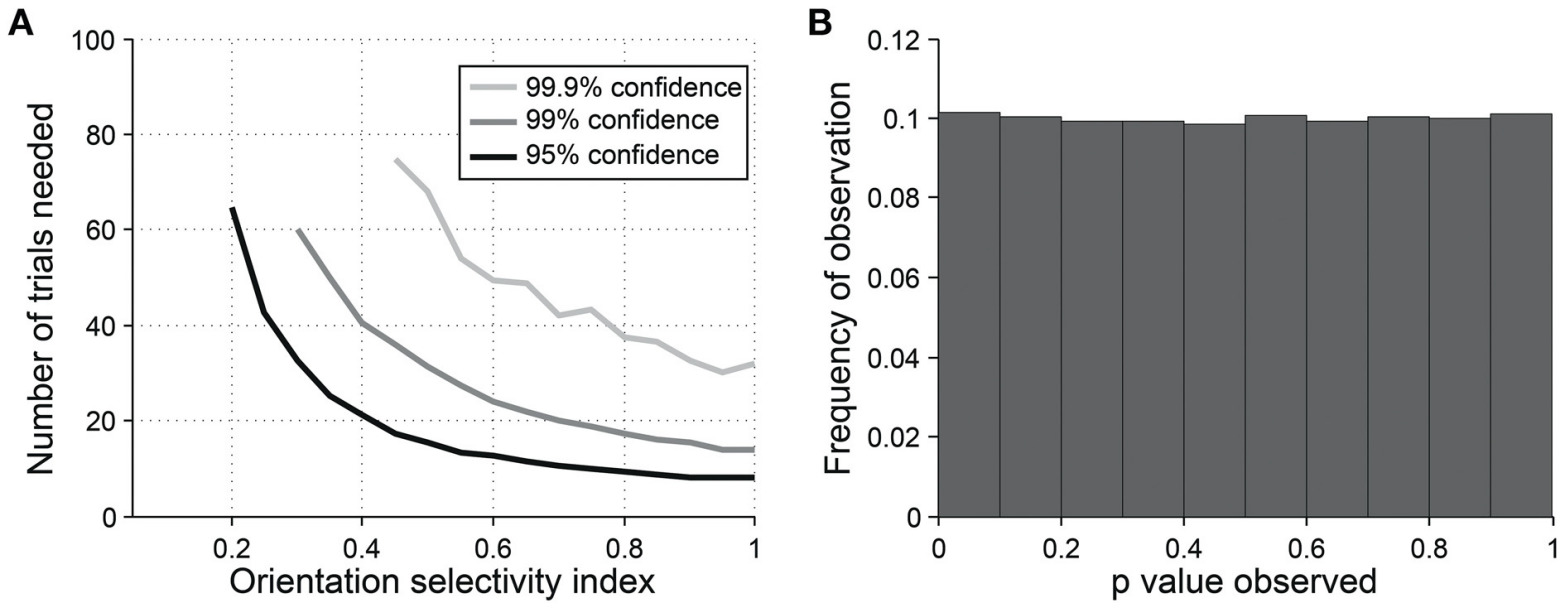
**Sensitivity and specificity of Hotelling's T-squared test for detecting orientation selectivity**. **(A)** Repeated simulations were performed with a single cell at different levels of underlying *OI* and different numbers of trials. 16 angles (22.5° steps) were used; noise = 40% at all conditions. Sensitivity of Hotelling's T-squared test was measured at three levels of significance: 95, 99, and 99.9%. For example, the black line shows the number of trials needed such that one would detect an *OI* difference of X with 95% confidence. **(B)** A cell was simulated with seven trials at underlying *OI* = 0. The simulation was repeated 200,000 times and each time a *p*-value was measured against H0: *OI* = 0. The frequency of observed *p*-values was uniform between 0 and 1, which is what would be expected for an unbiased test by repeated sampling of an unoriented cell.

For detecting orientation selectivity we use Hotelling's *T*^2^-test, which is a multivariate generalization of Student's *T*-test, to ask whether the 2-dimensional mean of orientation vectors is significantly different from [0,0] (Figure 7C). Figure 8 shows that this test reliably detects orientation selectivity. In this figure, a model cell is simulated at different levels of underlying *OI* and different numbers of trials. The figure shows the number of trials that would be needed to detect different *OI* levels vs. *OI* = 0 at three levels of sensitivity (95, 99, and 99.9%). The test is specific for orientation selectivity, with *p*-values distributed uniformly when *OI* = 0.

For detecting direction selectivity, it is possible in principle to apply Hotelling's *T*^2^-test to direction vectors. However, we have found that this method of testing for direction selectivity is quite insensitive because direction space is generally sampled too crudely to provide a reliable distribution of direction vectors. To address this problem, we developed a new test which we call the “direction dot product test.” This test uses both orientation vectors and direction vectors to assess the direction selectivity of a cell (Figure 7F).

In the direction dot product test, the first step is to obtain the orientation axis of the cell by calculating the angle of the average orientation vector. Next, we calculate the magnitude of the projection of each direction vector onto the orientation axis (this is what we call the “direction dot product” for each direction vector). This gives us a 1-dimensional distribution of direction dot product values, one value for each direction vector. Finally, Student's *T*-test is performed on the distribution of direction dot products with H0: mean = 0. The test yields a *p*-value for whether the average magnitude of a distribution of direction vectors is significantly greater than zero.

**Figure 9:**
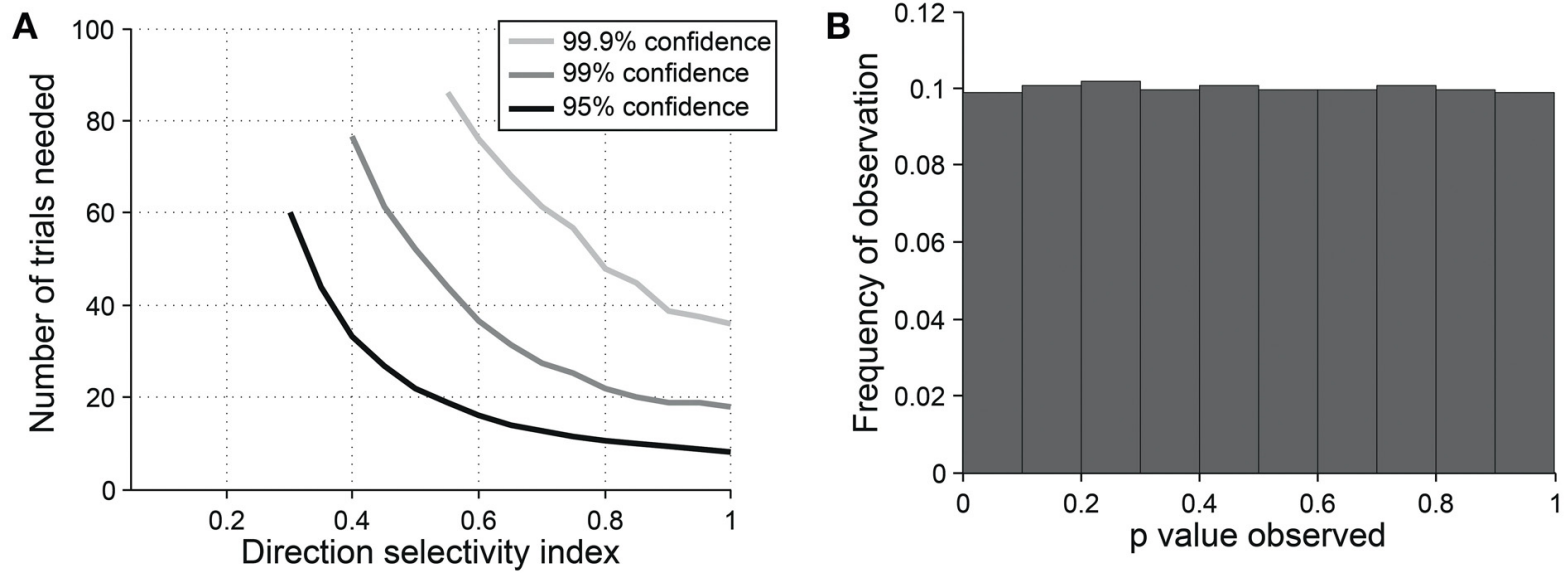
**Sensitivity and specificity of the direction dot product test for detecting direction selectivity**. **(A)** Repeated simulations were performed with a single cell at different levels of underlying *DI* and different numbers of trials; *OI* = 1 for all simulations. 16 angles (22.5° steps) were used; noise = 4 Hz at all conditions. Sensitivity of the direction dot product test was measured at three levels of significance: 95, 99, and 99.9%. **(B)** A cell was simulated with 7 trials at underlying *DI* = 0, *OI* = 1. The simulation was repeated 200,000 times and each time a *p*-value was measured against H0: *DI* = 0. The frequency of observed *p*-values was uniform between 0 and 1, which is what would be expected for an unbiased test by repeated sampling of cells that are indifferent to direction.

The direction dot product reliably detects direction selectivity. Figure 9 shows the direction dot product test applied to direction vectors from a simulated cell's response. The cell is simulated at different levels of underlying *DI* and different numbers of trials. The figure shows the number of trials that would be needed to detect different *DI* levels vs. *DI* = 0 at three levels of sensitivity (95, 99, and 99.9%). The test is specific for direction selectivity, with *p*-values distributed uniformly when *DI* = 0.

### Quantifying uncertainty and differences in orientation and direction preferences

Another objective that arises when one has a cell with selectivity for orientation/direction is to estimate the uncertainty of the measured selectivity parameters. Above we described tools for asking whether selectivity was significantly greater than zero. However, one might also like to obtain a measure of dispersion (e.g., standard deviation) or a confidence interval (e.g., standard error) for selectivity parameters. In principle, one could simply obtain this from the distribution of *OI*/*DI*/1-*OriCirVar*/1-*DirCirVar* values measured on repeated trials. However, these measures behave inconsistently as response properties vary, especially as selectivity approaches zero, so estimating the distribution of parameters or confidence intervals from them is not very meaningful. Fortunately, selectivity measures can be successfully employed to ask more specific statistical questions.

One common question, especially in the era of 2-photon imaging where many cells are recorded simultaneously, is to ask whether one population of cells has different average selectivity than another population (or, equivalently, whether a population recorded at one point in time has different average selectivity than the same population recorded at another point in time). The approach is simple: Measure selectivity coefficients from each cell in the two populations, and perform a 2-sample *T*-test to measure whether selectivity differs between them. The test can be performed using *OI*/*DI* or 1-*OriCirVar*/1-*DirCirVar* values. Table 1 shows results from simulations asking how many cells would be required to detect differences in underlying *OI* or *DI* at different levels of confidence. The table shows that, as seen above, circular variance performs better than *OI*/*DI*.

**Table 1:** **Minimum number of cells per condition that are needed to distinguish underlying orientation or direction selectivity index differences for two noise models**.

***OI* base + Δ**	**If readout is *OI***			**If readout is 1-*CirVar***		
	**0.95**	**0.99**	**0.999**	**0.95**	**0.99**	**0.999**
0.5 + 0.1	16/100	37/190	201/365	9/43	16/85	26/130
0.5 + 0.2	6/20	13/40	40/80	5/6	7/15	12/28
0.5 + 0.3	5/6	8/15	19/21	5/4	5/6	8/9
***DI* base + Δ**	**If readout is *DI***			**If readout is 1-*DirCirVar***		
	**0.95**	**0.99**	**0.999**	**0.95**	**0.99**	**0.999**
0.15 + 0.1	103/1k	183/2k	313/3k	38/181	69/324	117/519
0.15 + 0.2	26/145	48/265	74/410	10/40	18/64	29/112
0.15 + 0.3	11/45	21/80	34/145	5/12	9/23	15/40
0.3 + 0.1	84/291	152/521	233/950	30/80	56/147	92/250
0.3 + 0.2	20/58	39/109	64/170	9/20	15/36	24/61
0.3 + 0.3	10/22	18/41	31/75	5/10	8/14	13/21
0.5 + 0.1	76/131	141/241	218/384	26/43	43/76	82/140
0.5 + 0.2	20/30	37/50	59/110	8/10	13/20	22/31
0.5 + 0.3	10/11	17/20	27/35	5/5	7/10	12/14

The top three rows assume that the control group has an underlying OI that is 0.5, and the experimental group has the increment indicated in each row. The bottom rows assume that the control group has an underlying DI that is 0.15, 0.3, or 0.5 as specified, and the experimental group has the increment indicated in each row. The middle and right columns show the minimum cells that are needed to distinguish the control and experimental groups with a T-test at several confidence levels (0.95, 0.99, 0.999) if the experimenter is calculating OI (or DI) values as a readout (middle columns), or if the experimenter is calculating 1-CirVar (or 1-DirCirVar) values (right columns). The number of cells indicated is the number of cells required per condition (control or experimental), so twice this number would be required for a total experiment. Two noise models were used. In the data points to the left of the slash, 40% noise was added to the responses. In the data points to the right of the slash, “2-photon OGB-1AM” noise was used; that is, noise = 20% + 10 ^*^ response. Angle step size was 22.5°. Note that many fewer cells are needed to evaluate changes in orientation and direction selectivity if 1-CirVar or 1-DirCirVar is used as a readout as compared to OI or DI.

As an aside, one might wonder whether statistics on raw vectors could be used to answer this question. Since vector magnitudes correlate with selectivity, why not compare the vectors between the populations to see if selectivity has changed? The answer is that vector magnitudes, while they do correlate with selectivity, also correlate with tuning width and response magnitude (see Figure 3). If two populations differ in any of these response parameters, they will produce different vectors. Hence a test that looks for differences in vectors can give a positive result even if the populations are equally selective. Note that this effect isn't a problem when testing for the presence of selectivity, as we do in Figure 7, because here the null hypothesis is zero magnitude, which can only occur when selectivity is zero. Thus, statistics on raw vectors are suitable for detecting the presence of selectivity, but not for detecting differences in selectivity.

**Figure 10:**
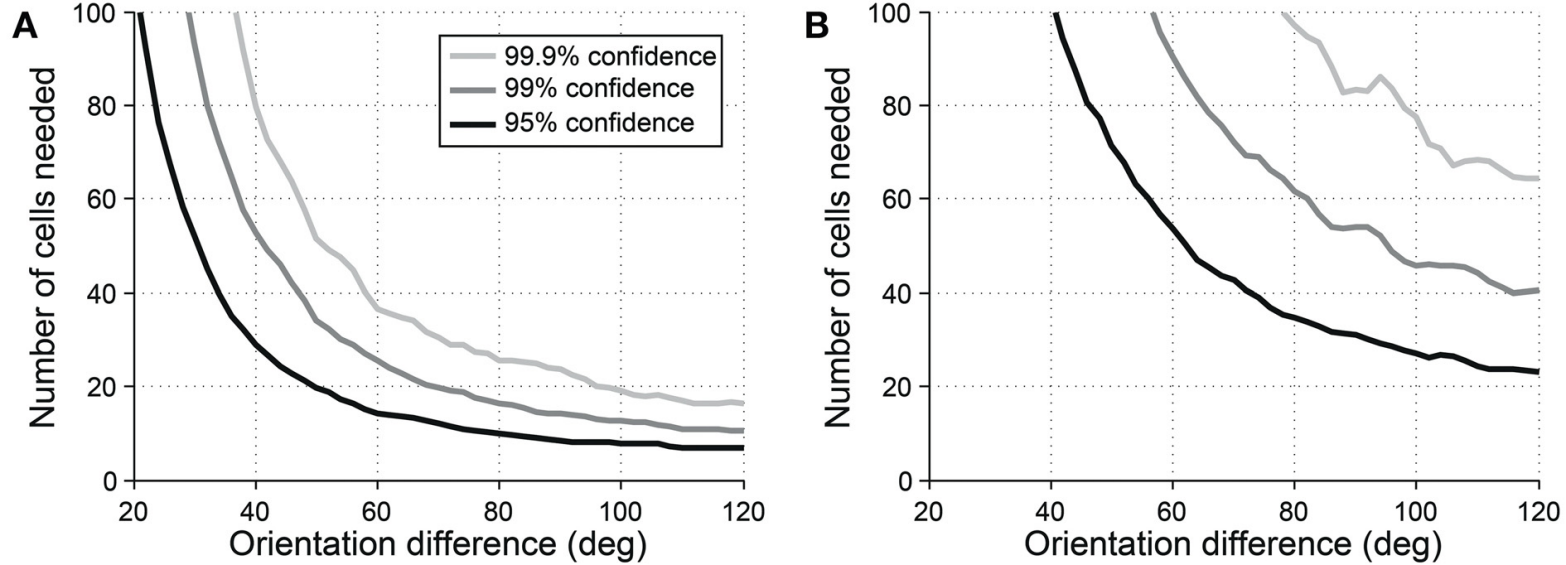
**Sensitivity of the 2-sample version of Hotelling's T-squared test for detecting differences preferred orientation between different cell populations**. Cells were simulated with seven trials each. We systematically varied the size of the cell populations and the size of the difference in preferred orientation. We measured the sensitivity for detecting the difference at three levels of confidence: 95, 99, and 99.9%. **(A)** Simulations performed using single-trial noise of 40% in all conditions. **(B)** Simulations performed using “2-photon OGB-1AM noise”: noise = 20 % + (10% × expected response).

Another common question is whether some specific response parameter differs between two cell populations. For example, one might wish to look for differences in preferred orientation between two populations. In this case, a vector-based test can be useful. Orientation vectors are affected by preferred orientation, so differences in preferred orientation lead to different distributions of vectors from the cells. Hotelling's *T*^2^-test (specifically the 2-sample version of the test, analogous to the 2-sample Student's *T*-test) can be used to detect such a difference. Figure 10 shows the sensitivity of the 2-sample Hotelling's *T*^2^-test in detecting differences in preferred orientation between two populations of cells.

However, this test must be used with caution. Vectors are affected by all response parameters including preferred orientation, tuning width, and response magnitude, so a positive result simply means that one or more of these parameters differs between the two populations; it cannot prove that the difference is in preferred orientation or any other single parameter. The test may be useful as a broad screen to detect generalized differences in response parameters. But if a difference in a specific response parameter is sought, the best method is to perform statistics with iterative fitting, as described below.

### Extracting parameters of orientation and direction tuning curves with fits

In order to address the question of how well a given population of neurons encodes the orientation or direction of a stimulus, it is often important to know the precise parameters of a cell's tuning function such as its tuning angle or tuning width. Previous work using Monte Carlo simulations (Swindale, 1998) found that the best method for estimating tuning parameters from orientation or direction responses is to fit these responses with a Gaussian curve. In orientation space, we can fit the responses using a single Gaussian:

$$R(\theta )=C+R_{p}e^{-\frac{ang_{ori}{\left(\theta -\theta_{pref}\right)}^{2}}{2\sigma^{2}}},$$

where *C* is a constant offset, θ_*pref*_ is the preferred orientation, *R_p_* is the above-offset response to the preferred orientation, ang_ori_(x) = min(x, x − 180, x + 180), wraps angular difference values onto the interval 0° to 90°, and σ is a tuning width parameter. If we wish to only analyze the portion of the response above the offset, then the tuning width (half-width at half-height) is equal to $\sqrt{\log4}\sigma$ (half-width at half height) (Carandini and Ferster, 2000).

In direction space, we can use a double Gaussian with the following equation:

$$R(\theta )=C+R_{p}e^{-\frac{ang_{dir}{\left(\theta -\theta_{pref}\right)}^{2}}{2\sigma^{2}}}+R_{n}e^{-\frac{ang_{dir}{\left(\theta +180-\theta_{pref}\right)}^{2}}{2\sigma^{2}}},$$

where *C* and θ_*pref*_ are defined as before, *R_p_* is the above-offset response to the preferred direction, *R_n_* is the above-offset response to the null direction, and ang_dir_(x) = min(x, x − 360, x + 360), wraps angular difference values onto the interval 0° to 180°, and σ is a tuning width parameter. Again, if we wish to only analyze the portion of the response above the offset, then the tuning width (half-width at half-height) is equal to $\sqrt{\log4}\sigma$ (half-width at half height) (Carandini and Ferster, 2000; Moore et al., 2005).

**Figure 11:**
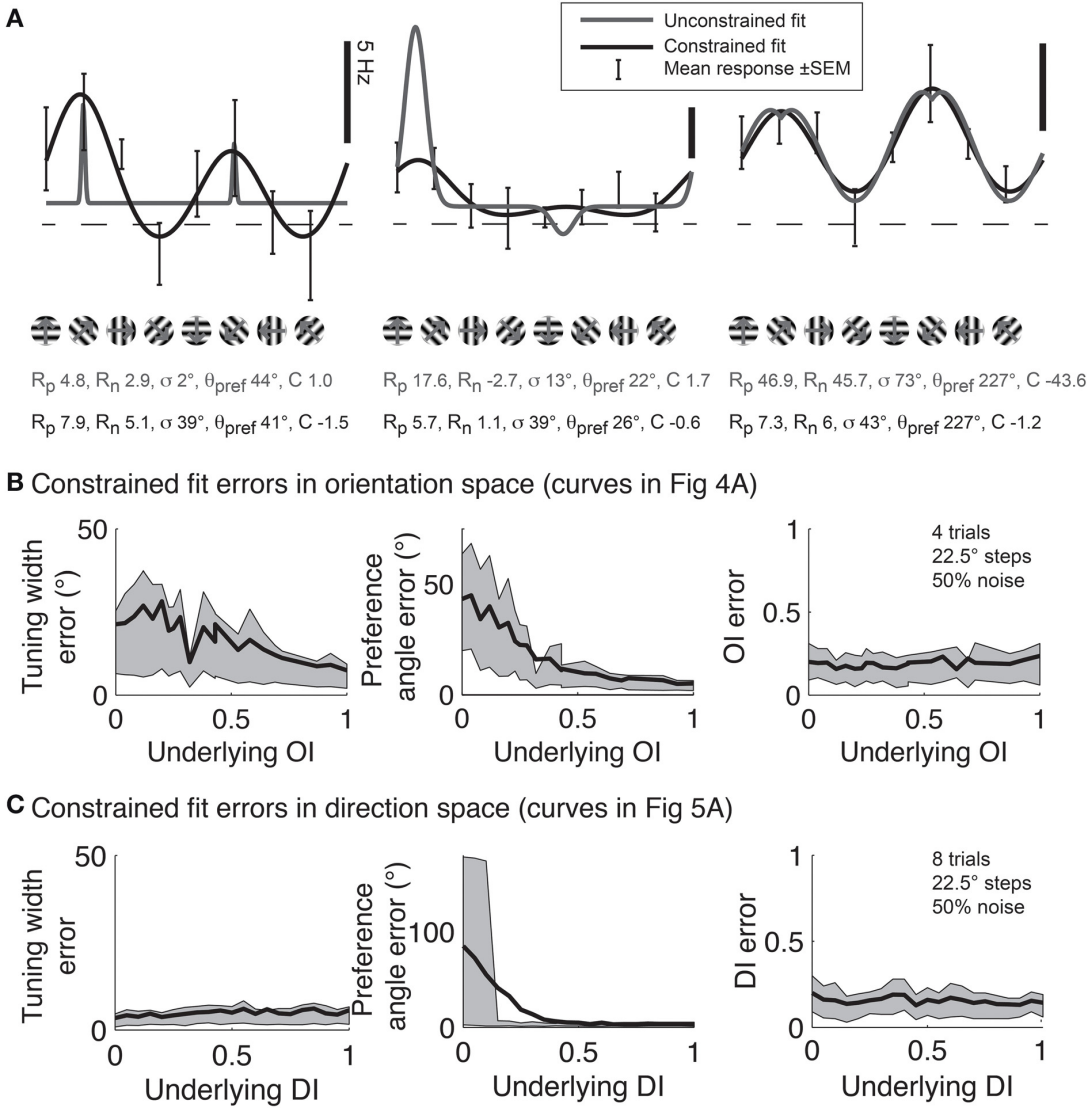
**Gaussian fits for assessing orientation and direction selectivity**. **(A)** Common errors with unconstrained fits (gray lines). Left: the unconstrained fit has gotten stuck in a local squared error minimum, using a tiny tuning width to fit 2 points very accurately. Middle: The unconstrained fit has used a peak response *R_p_* that is much larger than any point actually present in the data, and a physiologically implausible negative weight for the null direction. Right: The unconstrained fit has found a reasonable fit, but the parameters do not make physical sense. The unconstrained fit posits a constant offset that is highly negative, with large responses to the preferred and null directions. All of these fitting error can be solved by constraining the fit parameters to values that make physical sense (solid lines, see text). **(B)** Mean errors in tuning width, preferred angle, and *OI* for Monte Carlo simulations of cells with the underlying *OI*s in Figure 4. Gray patch indicates 25–75% interval **(C)** Mean errors in tuning width, preferred angle, and *OI* for Monte Carlo simulations of cells with the underlying *DI*s in Figure 5. Gray patch indicates 25–75% interval.

Although Gaussian fits are the best method for determining response parameters (Swindale, 1998), in practice there are several pitfalls to avoid. Several data analysis packages, such as Matlab (MathWorks) offer the ability to fit functions to data, but blindly applying a least squares fit to the data using the above functions often leads to poor fits. Common errors are described in Figure 11A. This problem is intractable in neural data because one never knows the “true” underlying response function, so it's impossible to say for certain that one fit is better than another. Hence, here we simulate responses with a known underlying response function, allowing us to evaluate the quality of our fits objectively.

To prevent poor fitting, we use 2 *ad-hoc* procedures. First, we provide several constraints on the fit parameters. We constrain the width parameter σto be at least as large as α/2, where α is the angle step used for stimulation; we force *C* to lie in the interval [-M,M], where M is the largest response to any stimulus; and *R_p_* and *R_n_* are constrained to lie in the interval [0, 3 M]. Second, we start the search using initial conditions that we expect will result in a good fit: θ_*pref*_ = θ_*M*_ where *M* = *R*(θ_*M*_), *R_p_* = *R_n_* =*M*, *C* = 0, and we explore several initial values for σ ≡ {α/2, α, 40°, 60°, 90°}. We take the fit with the lowest least square error for all these initial values of σ as the best fit of the data.

Using this *ad-hoc* fitting method, we can explore how well we can identify the tuning width and preferred angle for the same model cells we explored in Figure 4. The performance of the fit improves as the underlying *OI* increases (Figure 11B), although the error in angle preference is relatively large when *OI* is small. Because this error in angle preference is large when *OI* is small, we use another *ad-hoc* rule: we never report tuning widths or angle preferences from fits unless the data exhibits significant orientation selectivity by the Hotelling *T*^2^-test. The simulations of the model cells of varying direction selectivity in Figure 5 are fit with double Gaussian functions in Figure 11C. All of the tuning curves in Figures 5, 11B,C exhibit significant orientation selectivity, so the fit of tuning width and *DI* is excellent. As expected, when the underlying *DI* is smaller than about 0.25, the noise in the empirical responses obscures which of the two opposite directions along the preferred orientation axis is the “true” preferred direction.

**Figure 12:**
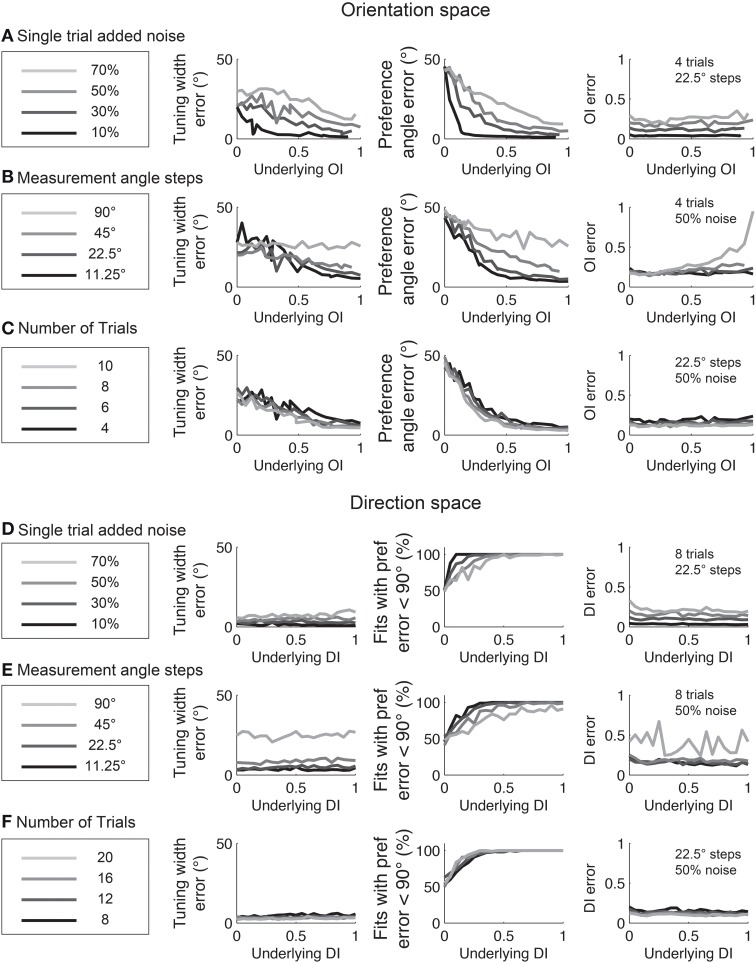
**The dependence of errors in identifying tuning width, preference angle, and *OI*/*DI* on neural noise and stimulus sampling**. On the Y axis of all plots is the median error between the “true” underlying quantity and the value provided by the fit. **(A–C)** are in orientation space, and **(D–F)** are in direction space. **(A,D)** Dependence of error on single trial noise as a percentage of the maximum response rate to the preferred direction. **(B,E)** Dependence of error on number of angle steps. Additional angle steps offer a modest improvement in estimating the fit parameters. **(C,F)** Dependence of error on the number of trials. More trials offer modest improvements in average accuracy.

The relationships between fit quality and noise and number of stimulus trials and stimulus angles are plotted in Figure 12. As with the circular variance index values, more angle steps are always better, but 22.5° step sizes provide relatively high quality fits when used with 6–8 trials.

### Quantifying uncertainty in fit parameters using iterative fits

Finally, one useful outcome of iterative fitting is that it can be used to estimate uncertainty in fit parameters and to do statistics on these parameters. The simplest method for doing this uses the Hessian matrix, which measures the steepness of the error function near the local minimum where the fit algorithm settles. The matrix is obtained by sampling the error function near the local minimum and measuring the partial second derivative of this function for each parameter; standard Matlab optimization tools produce the Hessian matrix as an output parameter. Once obtained, the Hessian matrix can be transformed to obtain standard errors of fit parameters, and these can then be used to perform statistics (Press et al., 1992).

Unfortunately, the Hessian method does not work for fitting orientation and direction curves. Since the Hessian matrix represents the second partial derivatives of the error function, it can only be obtained when the error function is reasonably smooth. As described above, achieving adequate fits of orientation and direction data requires strict constraints on the fitting procedure. Because of these constraints, the error function is not smooth and thus a meaningful Hessian matrix generally cannot be obtained when fitting orientation and direction curves.

Another method for using iterative fitting to quantify uncertainty in parameters is the bootstrap method. In this method, samples of data are repeatedly selected at random, with replacement, and fits are performed to each sample. The distribution of parameters obtained in these fits provides a reasonable estimate of the parameter distribution in the underlying population (Press et al., 1992), and hence this distribution can be used to calculate standard errors and to do statistics.

**Figure 13:**
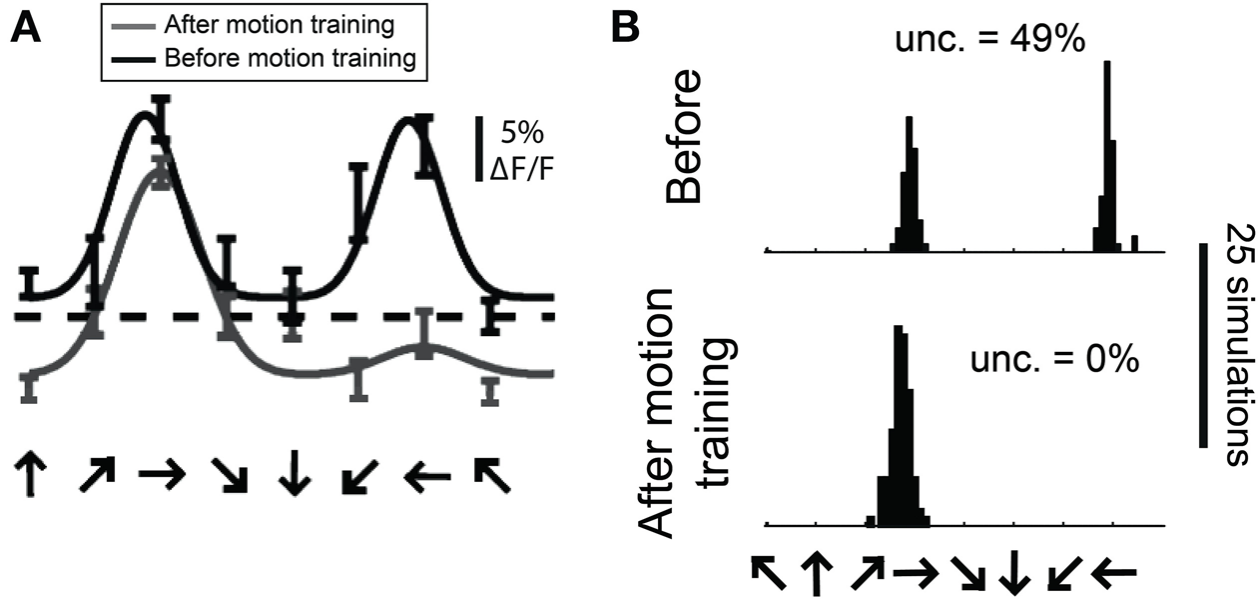
**Estimating the distribution of preferred direction using bootstrap methods. (A)** Data from a cell recorded with 2-photon calcium imaging using OGB-1AM before and after extended exposure to a motion stimulus (“motion training”). Bars show ±1 standard error of the response. Solid lines show best fits with double Gaussian functions. The dashed line indicates mean response to a gray screen. **(B)** Distribution of preferred directions in bootstrap simulations of the cell shown in **(A)**. The data from this cell was randomly resampled with replacement, creating a “simulated” cell, and this simulated data set was fit with double Gaussian functions. This procedure was repeated 100 times in each training condition, yielding the observed distributions of preferred direction. “Unc.” lists the preference uncertainty, meaning the percentage of simulations whose preferred direction differed from the mean direction by more than 90°.

In a previous study we employed the bootstrap method to estimate the distribution of preferred direction in individual cells from 2-photon recordings before and after extended exposure to a motion stimulus (Figure 13, modified from Li et al.). To obtain a distribution of preferred direction, we used the *N* trials obtained from a cell and created a “simulated” cell by randomly resampling these trials *N* times with replacement. The simulated data was then fit with a double Gaussian as described above. This procedure was repeated 100 times and the preferred direction was obtained for each simulation, yielding a distribution of preferred direction values from this cell.

One way we used this distribution was to detect significant direction selectivity. We quantified the “uncertainty” in direction preference, which is the percentage of simulations whose preferred direction differed from the mean preferred direction by more than 90°. This uncertainty can vary between 0 and 50%, so we interpret (*uncertainty* × 2) as a *p*-value for significant direction selectivity. We found that the sensitivity and specificity of this method for detecting direction selectivity is similar to what we obtain with the dot product direction test described above.

There are several drawbacks to the bootstrap method. First, the method is very computationally intensive, with a standard test requiring several days of computer time. More importantly, results obtained from the bootstrap method depend on a variety of factors that are not related to the data. Specifically, the outcome of the test depends on the fitting algorithm employed, the initial value used in the fit, and the constraints placed on the fit. Researchers using the bootstrap method must take care to record and publicize details about their technique so that others may reproduce their findings.

An alternative approach that generates confidence intervals is a Bayesian approach, such as that described in Cronin et al. (2010). The authors develop methods for estimating the entire probability distribution of each parameter value.

## Discussion

Orientation and direction tuning are probably the most intensively studied response properties in the cortex. Historically, these studies have focused on cells with strong selectivity as determined by simple comparisons between preferred and non-preferred responses; cells without such obvious selectivity were often declared, simply, “unselective.” However, the advent of advanced techniques for recording and manipulating neurons requires us to investigate subtle differences between cells and to extend our analysis to cells with low selectivity. We need statistical tools that are suitable for addressing these subtle questions.

Traditional measures for quantifying orientation and direction selectivity rely on assigning the stimulus evoking the strongest response as the “preferred” stimulus for the cell and assign the opposite stimulus as “non-preferred.” The most commonly-used measures, *OI* and *DI*, compare the strongest stimulus to orthogonal stimuli (for *OI*) or opposite-direction stimuli (for *DI*). Our analysis shows that these measures are generally unreliable, especially for cells that have low selectivity or high noise (Figures 2, 4). The key flaw with *OI*/*DI* and related measures is that preferred and non-preferred stimuli are always taken from sampled values of orientation/direction; if the true preferred stimulus lies between sampled values (which is likely to be the case), it will be missed.

To obtain an accurate estimate of preferred and non-preferred stimuli, one must extrapolate between measured values. Vector-based methods effectively extrapolate measured responses by calculating the vector average of responses on each trial. Specifically, for quantifying selectivity, we recommend 1-*OriCirVar* (for orientation) and 1-*DirCirVar* (for direction). These measures demonstrate greater reliability than *OI*/*DI* (Figures 4, 5) and they are more sensitive than *OI*/*DI* for detecting differences in selectivity between two populations (Table 1).

Vectors can also be used to assess whether a cell's selectivity is statistically significant. In this approach, we ask whether the 2-dimensional mean of orientation or direction vectors is significantly from zero. Specifically, Hotelling's *T*^2^-test on orientation vectors is reliable for detecting orientation selectivity (Figure 8) and the direction dot product test is reliable for detecting direction selectivity (Figure 9).

In some cases, we need to probe beyond selectivity to ask about specific response parameters such as tuning angle or tuning width. Vectors can be used to detect differences in these parameters between two populations (Figure 10); however, vector-based methods cannot identify which particular parameter or parameters are responsible for the difference. To answer such precise questions, we recommend another method of extrapolation: fitting data with Gaussian curves (for orientation) or double Gaussian (for direction). Swindale (1998) showed that least squared fitting with these functions provided the best method for extracting response parameters from orientation and direction data. This method provides accurate estimates of response parameters for cells with significant selectivity, provided that the fitting routine is appropriately constrained to avoid erroneous local minima (Figures 11, 12).

Fitting also offers a tool for estimating the uncertainty of response parameters via the bootstrap method, where the data is randomly resampled multiple times with replacement and fits are performed to the resampled data. This method generates a distribution of values for each parameter which serves as an accurate estimate of the true distribution (Figure 12). Hence this method allows precise statistical questions to be asked about each response parameter underlying a cell's response. Note that alternative methods for fitting data and estimating parameters have been used (e.g., Cronin et al., 2010); we have not compared these methods to those described here.

Table 2 summarizes our recommendations for which method is best suited to a variety of quantitative questions regarding cells with orientation and direction tuning. Our goal is to provide tools for researchers to ask more refined statistical questions than have been possible using traditional measures such as *OI*/*DI*. As research advances into the precise mechanisms underlying orientation and direction tuning, robust quantitative methods will be required to distinguish competing theories. We hope the tools presented here will help accomplish this goal.

**Table 2:** **Recommended methods for answering several common scientific questions involving orientation and direction selectivity**.

**Question**	**Recommended method**
Quantifying the degree of orientation selectivity	1-*OriCirVar*
Quantifying the degree of direction selectivity	1-*DirCirVar*
Testing for significance of orientation selectivity	Hotelling's *T*^2^-test on orientation vectors
Testing for significance of direction selectivity	Direction dot product test on direction vectors
Comparing orientation selectivity between two populations	2-sample Student's *T*-test on 1*-OriCirVar* values
Comparing direction selectivity between two populations	2-sample Student's *T*-test on 1-*DirCirVar* values
Screening for any difference in response parameters (e.g., preferred orientation, tuning width, peak height) between two populations	2-sample Hotelling's *T*^2^-test on orientation vectors
Extracting response parameters such as tuning angle or tuning width	Fit data with Gaussian (for orientation data) or double Gaussian (for direction data)
Quantifying the confidence/uncertainty of response parameters such as tuning angle or tuning width	Bootstrap method: Resample data with replacement, then fit resampled data with Gaussian (for orientation data) or double Gaussian (for direction data)

## Author contributions

Mark Mazurek, Marisa Kager, and Stephen D. Van Hooser performed analysis, Mark Mazurek and Stephen D. Van Hooser wrote the paper with comments from Marisa Kager.

### Conflict of interest statement

The authors declare that the research was conducted in the absence of any commercial or financial relationships that could be construed as a potential conflict of interest.
